# Genetic variability and high proportion of HIV-1 BF1 recombinant strains among vertically infected children in São Paulo, Brazil

**DOI:** 10.1186/1742-4690-9-S1-P4

**Published:** 2012-05-25

**Authors:** Ana Carolina Soares de Oliveira, Antonio Charlys da Costa, Vanessa Pouza Martinez, Maria Teresa Maidana, Giret Regina Celia de Menezes Succi, Ester Cerdeira Sabino Esper, Georges Kallas, Sabri Saeed Sanabani

**Affiliations:** 1 São Paulo Institute of Tropical Medicine, São Paulo, Brazil

## Introduction

The enormous genetic variability of human immunodeficiency virus type 1 (HIV-1) continues to present a major challenge for vaccine design and frustrate efforts to halt the epidemic. A proper understanding of this phenomenon is a prerequisite for proper epidemiology, genetic diagnosis, and successful drugs and vaccines. In this study, we undertook a detailed molecular epidemiological investigation on HIV-1 vertically-infected children born from 1993 to 2008 in the state of São Paulo, Brazil.

## Material and methods

HIV-1 proviral DNA was extracted from the peripheral blood mononuclear cells of 48 participants. The near full-length genomic (NFLG) and partial fragments were determined by overlapping nested PCR and direct sequencing. The data were phylogenetically analyzed.

## Results

Of the 48 samples (median age 11.8 years, range 4-20.6 years) studied, 3 (6.2%) NFLGs and 39 (81.2%) partial fragments were successfully subtyped. Of the successfully subtyped sequences, 20 (47.6%) were subtype B sequences, 17 (40.4%) BF1 recombinants, and 5 (11.9%) subclade F1. Two of the partial BF1 chimeric isolates shared an identical recombination structure. Predictions of viral tropism using the computer program geno2pheno [co receptor] for phenotype prediction were determined for 15 subjects. X4 or X4 dual or mixed-tropic viruses were seen in 3 (20%) of participants and the V3 sequences of 12 patient virus strains (80%) were predicted to be R5-tropic virus.

## Conclusions

Our data provided evidence of unexpectedly high proportion of BF1 recombinants viruses transmitted from the first mother-to-child since the earliest days of the epidemic to the present time in Brazil. These findings offers additional insights to understanding the diversity of HIV-1 strains currently circulating in Brazil, with future implications for diagnosis, therapy, and efficient vaccine development.

